# Day-4 antipseudomonal and anti-MRSA de-escalation was not associated with higher mortality in critically ill patients with sepsis

**DOI:** 10.3389/fmed.2026.1924203

**Published:** 2026-08-27

**Authors:** Yuhang Zhang, Guoying Liao, Jianli Song, Yinsheng Liao, Lulu Zhang

**Affiliations:** 1 Department of Anesthesiology, The Second Affiliated Hospital of Chongqing Medical University, Chongqing, China; 2 Department of Anesthesiology, Zigong Fourth People's Hospital, Zigong, Sichuan, China

**Keywords:** antibiotic de-escalation, antimicrobial stewardship, antipseudomonal antibiotics, intensive care unit, MIMIC-IV, overlap weighting, sepsis, target trial emulation

## Abstract

**Background:**

Broad empiric antibiotics are often appropriate at the start of intensive care unit (ICU) sepsis care; however, continued antipseudomonal or anti-methicillin-resistant *Staphylococcus aureus* (MRSA) coverage may no longer be justified by the day-4 reassessment. We examined antibiotic de-escalation at this clinical decision point.

**Materials and methods:**

We emulated two parallel target trials in Medical Information Mart for Intensive Care IV (MIMIC-IV) v3.1 and conducted a supportive multicenter analysis in the eICU Collaborative Research Database (eICU-CRD) v2.0. The strict MIMIC-IV cohort included patients with ICU sepsis, early receipt of broad-spectrum antibiotics, survival and continued observation through 96 h, available culture information before time zero, no recorded multidrug-resistant organism evidence, and no prespecified exclusions. Time zero was 72 h after antibiotic initiation; exposure was classified during 72–96 h, and follow-up began at 96 h. Trial A compared anti-MRSA de-escalation with continuation, and Trial B compared antipseudomonal de-escalation with continuation. Propensity scores, overlap weighting, and bootstrap confidence intervals were used.

**Results:**

The strict MIMIC-IV cohort included 5,762 patients. In Trial B, 1,569 patients were de-escalated, and 3,070 continued antipseudomonal coverage. De-escalation was associated with lower 30-day mortality (24.1% vs. 29.0%; risk difference, −4.91 percentage points; 95% confidence interval, −7.27 to −2.23), lower in-hospital death, lower stage 2–3 acute kidney injury, and more death-adjusted antibiotic-free days. In Trial A, 3,041 patients were de-escalated, and 1,728 continued anti-MRSA coverage; there was no evidence of higher 30-day mortality (26.3% vs. 27.8%; risk difference, −1.44 percentage points; 95% confidence interval, −3.75 to 1.11), and de-escalation was associated with more antibiotic-free days. In eICU-CRD, Trial B showed a directionally consistent association with lower hospital mortality (10.2% vs. 12.7%; cluster-bootstrap risk difference, −2.42 percentage points; 95% confidence interval, −3.87 to −0.87).

**Conclusion:**

Day-4 de-escalation was not associated with higher mortality and was accompanied by reduced antibiotic exposure. The lower mortality and kidney-injury estimates in Trial B are exploratory because de-escalation may mark early recovery that is incompletely captured in structured data. The eICU-CRD analysis provides directional support rather than formal external validation. Prospective evaluation is warranted.

## Introduction

Early empiric broad-spectrum antibiotics are central to the initial treatment of sepsis in the intensive care unit (ICU). This practice is clinically understandable: early sepsis care often begins before the infection source, pathogen, and susceptibility profile are known. The same uncertainty that justifies broad coverage at presentation becomes less acceptable after cultures, laboratory trends, and the first 72 h of clinical response have been observed. Contemporary sepsis guidance therefore places antimicrobial stewardship alongside timely treatment and encourages daily reassessment of antibiotic spectrum and duration (1). This reassessment also matters beyond the individual patient, because unnecessary broad-spectrum exposure contributes to selection pressure for antimicrobial resistance, one of the leading global threats to health (2).

The practical question for ICU clinicians is not whether broad empiric therapy should be withheld at presentation. Rather, it is whether coverage against methicillin-resistant *Staphylococcus aureus* (MRSA), *Pseudomonas aeruginosa*, or other resistant gram-negative organisms can be stopped or narrowed when no multidrug-resistant organism (MDRO) has been identified by the day-4 review. Empiric anti-MRSA and antipseudomonal therapy are common; however, de-escalation remains variable, and resistant pathogens are identified in only a minority of patients initially receiving broad coverage (3–5). A recent target trial emulation in 36,924 patients with community-onset sepsis reported similar 90-day mortality after day-4 anti-MRSA or antipseudomonal de-escalation, together with fewer antibiotic days and shorter hospitalization (5). Randomized and stewardship studies similarly support reducing unnecessary exposure but do not suggest that de-escalation itself confers a survival benefit (6, 7). ICU-specific evidence remains limited, and treatment selection may be particularly sensitive to early physiologic recovery and bedside clinical judgment.

We, therefore, used target trial emulation, an approach that specifies the trial one would ideally conduct before mapping it to observational data, to study a defined day-4 ICU decision (8–10). MIMIC-IV was used for the strict primary analysis because it provides detailed time-stamped medication, microbiology, laboratory, and ICU treatment data. eICU-CRD was used for a supportive multicenter analysis of directional consistency; its microbiology data are not sufficiently complete to recreate the strict MIMIC-IV no-MDRO cohort. The main clinical question was whether de-escalation was associated with evidence of harm and with reduced antibiotic exposure, while lower mortality or acute kidney injury estimates were treated as exploratory signals that could reflect residual confounding by recovery.

## Materials and methods

### Study design and data sources

We conducted a retrospective cohort study using target trial emulation and reported it with attention to guidance for observational studies and target trial emulation (8, 11). The primary analysis used MIMIC-IV v3.1, a deidentified electronic health record database from Beth Israel Deaconess Medical Center that includes hospital and ICU records from 2008 through 2022 (12). The supportive multicenter analysis used the eICU Collaborative Research Database (eICU-CRD) v2.0, a US ICU database from 2014 to 2015 (13).

The target decision was whether to de-escalate or continue the relevant broad-spectrum coverage at approximately day 4 after the first qualifying broad-spectrum antibiotic. Time zero was set at 72 h after initiation of the first qualifying broad-spectrum antibiotic. A 24 h grace period allowed clinical orders and administration records to reflect the day-4 decision. Exposure was classified during 72–96 h, and outcome follow-up began at the 96-h landmark. Separating exposure classification from follow-up reduced immortal time bias.

### Eligibility criteria

**Figure 1:**
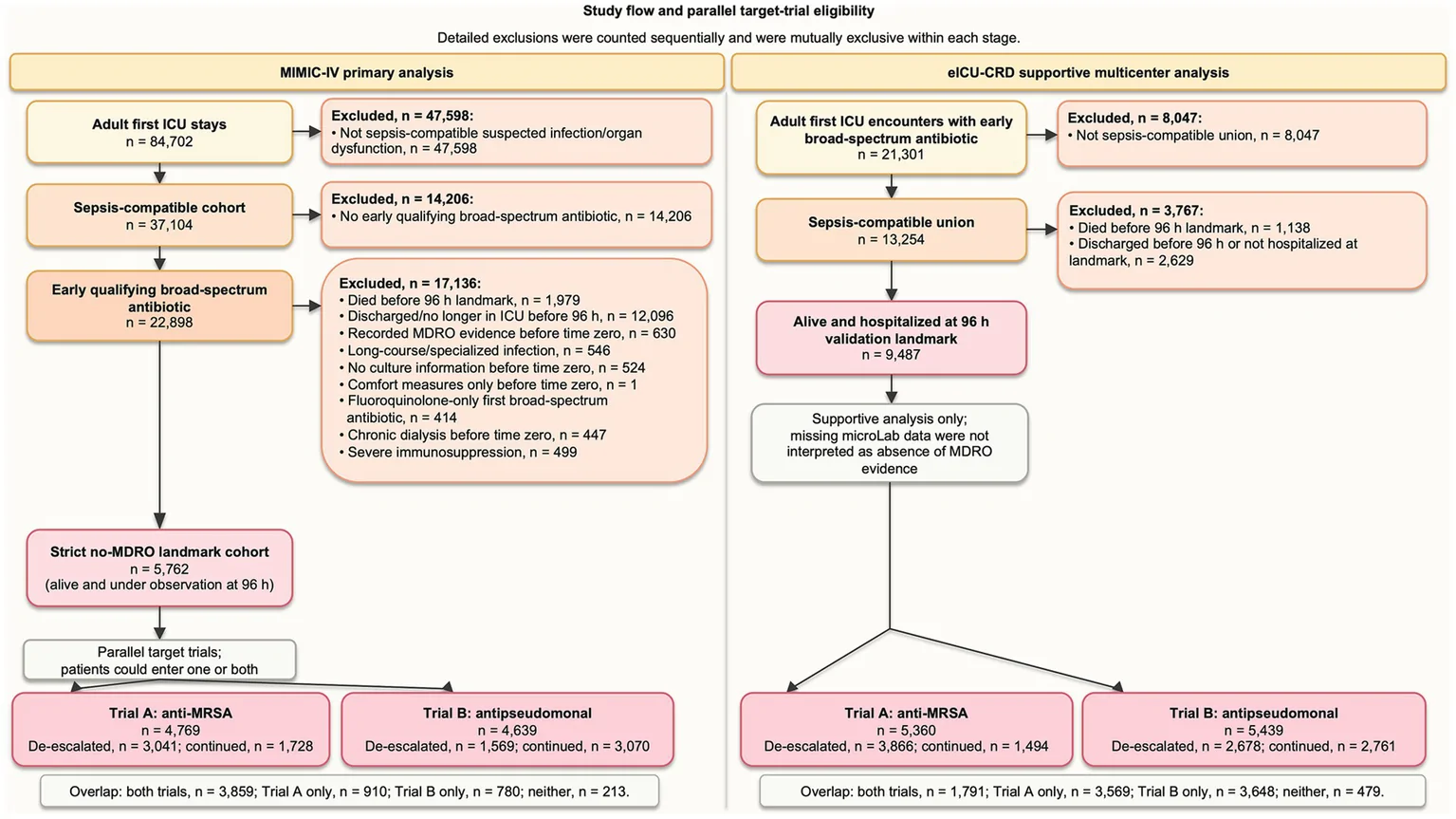
Study flow and parallel target-trial eligibility. Exclusion reasons were applied sequentially and were mutually exclusive for cohort accounting; individual criteria could coexist clinically. Trial A and Trial B were emulated in parallel, and patients could enter one or both trials. The eICU-CRD panel represents a supportive multicenter analysis because microLab data were sparse; missing microLab data were not interpreted as absence of MDRO evidence. ICU, intensive care unit; MDRO, multidrug-resistant organism; MRSA, methicillin-resistant *Staphylococcus aureus*.

In MIMIC-IV, eligible patients were adults admitted to the ICU with sepsis-compatible suspected infection and organ dysfunction and an early qualifying broad-spectrum antibiotic initiated between 6 h before and 24 h after ICU admission. Patients had to remain alive and under observation through the 96-h landmark, have culture information available before time zero, and have no recorded MDRO evidence before time zero. Sepsis was operationalized using suspected infection paired with organ dysfunction, which was consistent with electronic health record applications of Sepsis-3 concepts (14, 15). The first ICU stay was used to reduce within-patient dependence. We included all eligible first ICU stays that met these criteria (complete enumeration of the eligible population); no probability sampling was applied, and Figure 1 shows the full sequential accounting.

From the early broad-spectrum antibiotic cohort, patients were excluded sequentially if they died before the 96-h landmark; were discharged or no longer under ICU observation before 96 h; had recorded MDRO evidence before time zero; had an infection usually requiring prolonged or specialized antimicrobial therapy; lacked culture information before time zero for strict no-MDRO classification; had comfort-measures-only status before time zero; received a fluoroquinolone-only first qualifying broad-spectrum antibiotic exposure; had chronic dialysis before time zero; or had severe immunosuppression. Long-course or specialized infections included, when identifiable, endocarditis, osteomyelitis, central nervous system infection, necrotizing soft tissue infection, complex prosthetic infection, severe burn infection, and persistent bacteremia. Each excluded patient was assigned to the first applicable reason, making the reason-specific counts mutually exclusive within that stage.

In eICU-CRD, adult first ICU encounters with early broad-spectrum antibiotic exposure were screened using a sepsis-compatible union based on available culture, diagnosis, treatment, and severity information. Encounters had to be alive and hospitalized at the 96-h landmark; those who died or were discharged before the landmark were excluded. Because microLab records were sparse, the strict MIMIC-IV culture-availability and no-MDRO criteria were not imposed, and missing microLab data were not interpreted as absence of MDRO evidence. The eICU-CRD analysis was therefore designed to assess directional consistency in a multicenter setting rather than to provide formal external validation of the strict MIMIC-IV cohort. Exclusion reasons in Figure 1 were counted sequentially and were mutually exclusive within each stage.

### Treatment strategies

Trial A evaluated de-escalation of systemic anti-MRSA coverage. Anti-MRSA agents included intravenous vancomycin, linezolid, daptomycin, and ceftaroline. Oral vancomycin used for *Clostridioides difficile* infection was not counted as systemic anti-MRSA therapy. De-escalation was defined as discontinuation of systemic anti-MRSA coverage during the grace period without replacement by another systemic anti-MRSA agent. Continuation was defined as ongoing anti-MRSA coverage beyond the grace period.

Trial B evaluated de-escalation of antipseudomonal or resistant gram-negative coverage. Antipseudomonal agents included piperacillin-tazobactam, cefepime, ceftazidime, aztreonam, meropenem, imipenem-cilastatin, doripenem, newer resistant gram-negative agents, aminoglycosides, and ciprofloxacin or levofloxacin when used as therapeutic antipseudomonal coverage. De-escalation was defined as stopping the relevant coverage or switching to a narrower agent, such as ceftriaxone, cefazolin, ampicillin-sulbactam, ampicillin, penicillin, amoxicillin-clavulanate, or an oral step-down agent. Continuation was defined as ongoing same-spectrum or broader antipseudomonal coverage beyond the grace period.

Trial A and Trial B were emulated in parallel. Patients could enter one or both trials according to the broad-spectrum classes received during early treatment; therefore, the trial cohorts were not mutually exclusive, and their sizes were not expected to sum to the overall landmark cohort. Each trial was analyzed separately using the same design and adjustment strategy. Because some patients contributed to both trials, estimates across Trial A and Trial B were not treated as statistically independent, and no formal between-trial comparison was performed.

### Outcomes

In MIMIC-IV, the primary mortality outcome was recorded as all-cause death within 30 days after the 96-h landmark, regardless of discharge status. In-hospital death was defined as death during the index hospitalization; consequently, 30-day mortality could exceed in-hospital mortality when deaths after discharge were captured. Other outcomes were creatinine-based Kidney Disease: Improving Global Outcomes (KDIGO) stage 2–3 acute kidney injury (AKI) during the 7 days after the landmark and death-adjusted antibiotic-free days (AFDs) through days 14 and 28 (16). Death-adjusted AFDs assigned zero days to patients who died before the relevant horizon. Patients receiving chronic dialysis before time zero were excluded from the strict cohort. Pre-landmark AKI did not by itself exclude eligibility, and renal measurements before time zero were included in the propensity score models. Death was not modeled as a competing event for AKI. Restart of the same broad-spectrum class after de-escalation was evaluated only within the de-escalated group as a descriptive safety indicator and was not compared with continuation.

In eICU-CRD, the outcome for the supportive analysis was hospital mortality. Longer-term mortality, microbiology-linked outcomes, and kidney outcomes were not considered sufficiently comparable across hospitals for replication. Missing microLab records were not interpreted as negative culture results.

### Covariates and statistical analysis

Propensity score models included information available before time zero. Covariate domains included age, sex, race or ethnicity, admission type, ICU type, calendar period, comorbid conditions, suspected infection timing, culture information available before time zero, initial antibiotic regimen, organ dysfunction measures, mechanical ventilation, vasopressor use, lactate, mean arterial pressure, temperature, white blood cell count, creatinine, urine output, and other laboratory and physiologic summaries. Dynamic summaries were constructed over early windows before time zero, including recent, worst, and change measures where available.

The primary adjustment used propensity scores and overlap weighting. Overlap weighting emphasizes patients for whom either strategy was clinically plausible and limits the influence of extreme weights (17, 18). Covariate balance was assessed using absolute standardized mean differences (SMDs), with values below 0.10 considered acceptable.

Binary outcomes were summarized as weighted risks and risk differences (RDs) in percentage points. Continuous AFD outcomes were summarized as weighted means and mean differences (MDs) in days. Confidence intervals (CIs) were estimated by bootstrap procedures that re-estimated propensity scores and weights in each replicate. MIMIC-IV analyses used 500 bootstrap replicates. The eICU-CRD analysis used 300 replicates, with a hospital-cluster bootstrap for the primary uncertainty estimate.

Sensitivity analyses in MIMIC-IV included alternative exposure definitions, exclusion of cases with final-MDRO-discordant results, restriction to culture-negative patients, analysis of patients with recorded non-MDRO-positive results, restriction to patients whose relevant microbiology result was recorded before time zero (using the structured result-entry timestamp, storetime, as a proxy for result availability), and exclusion of temperature variables from the propensity score model. eICU-CRD analyses included models without hospital identifiers, hospital-cluster bootstrap, exclusion of fluoroquinolone-only first broad-spectrum antibiotic exposure, and leave-one-hospital-out analyses for Trial B. Covariate balance and propensity score distributions for models without hospital identifiers are provided in Supplementary Figures 2–5. The results are presented as estimates with 95% CIs; *p*-values were not used as the basis for interpretation. Effective sample sizes after overlap weighting were calculated using the final overlap weights. Because the study was designed around prespecified safety outcomes with exploratory secondary and sensitivity analyses, no formal multiplicity adjustment was applied. Secondary mortality, kidney, and sensitivity estimates were interpreted as exploratory rather than confirmatory.

### Ethics

MIMIC-IV and eICU-CRD contain deidentified data and are available to credentialed investigators under data use agreements. This secondary analysis involved no patient contact or attempts at reidentification; formal ethics approval and individual informed consent were not required.

## Results

### Cohort assembly

Cohort assembly for both databases is shown in Figure 1. In MIMIC-IV, 84,702 adult first ICU stays were screened; 37,104 met the sepsis-compatible definition, and 22,898 received an early qualifying broad-spectrum antibiotic. Before formation of the strict cohort, 17,136 patients were excluded, most commonly because they were discharged or no longer under ICU observation before 96 h (*n* = 12,096) or died before the landmark (*n* = 1,979). The remaining prespecified exclusions are detailed in Figure 1. The strict no-MDRO landmark cohort included 5,762 patients. Of these, 3,859 were eligible for both parallel trials, 910 for Trial A only, 780 for Trial B only, and 213 for neither trial. Trial A included 4,769 patients, of whom 3,041 were classified as anti-MRSA de-escalated and 1,728 as continued anti-MRSA coverage. Trial B included 4,639 patients, of whom 1,569 were classified as antipseudomonal de-escalated and 3,070 as continuing antipseudomonal coverage.

In eICU-CRD, 21,301 adult first ICU encounters with early broad-spectrum antibiotic exposure were screened; 13,254 met the sepsis-compatible union definition. Before the 96-h validation landmark, 1,138 encounters were excluded because the patient died and 2,629 because the patient was discharged or was otherwise not hospitalized at the landmark, leaving 9,487 encounters. Of these, 1,791 were eligible for both trials, 3,569 for Trial A only, 3,648 for Trial B only, and 479 for neither trial. Trial A included 5,360 patients, with 3,866 classified as de-escalated and 1,494 as continuing anti-MRSA coverage. Trial B included 5,439 patients, with 2,678 classified as de-escalated and 2,761 as continuing antipseudomonal coverage.

### Baseline characteristics and balance

**Figure 2:**
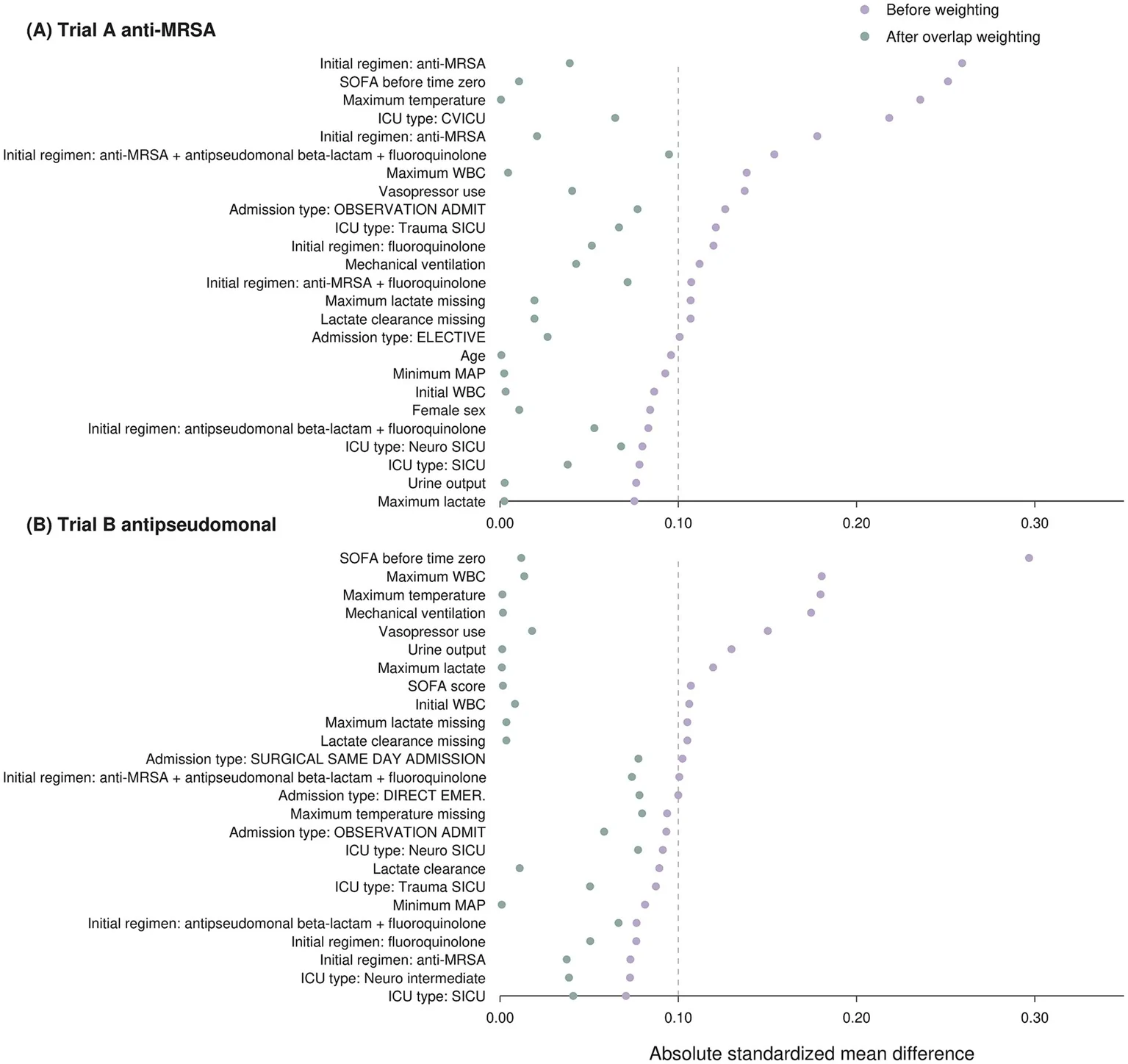
Covariate balance before and after overlap weighting in the MIMIC-IV target trials. **(A)** Trial A, anti-MRSA de-escalation. **(B)** Trial B, antipseudomonal de-escalation. The dashed line denotes an absolute standardized mean difference of 0.10. MRSA, methicillin-resistant *Staphylococcus aureus*; SMD, standardized mean difference.

Before weighting, continued-coverage patients generally had greater illness severity than de-escalated patients. In Trial B, the continued group had higher Sequential Organ Failure Assessment (SOFA) scores before time zero, more mechanical ventilation and vasopressor use, higher maximum white blood cell count and temperature, and lower urine output. After overlap weighting, measured clinical covariates were well balanced. In the core MIMIC-IV baseline table, the maximum absolute SMD after overlap weighting was 0.043 for Trial A and 0.029 for Trial B (Table 1; Figure 2). Major comorbidities, early antibiotic-class patterns, and time-zero-visible microbiological characteristics for the strict cohort are summarized in Supplementary Tables 1, 3.

**Table 1:** Core MIMIC-IV baseline characteristics before and after overlap weighting.

Trial	Covariate	De-escalated, unweighted	Continued, unweighted	De-escalated, overlap weighted	Continued, overlap weighted	Weighted SMD
Trial A anti-MRSA	Age	65.03 (15.70)	63.50 (16.14)	64.16 (15.91)	64.17 (15.97)	0.001
Trial A anti-MRSA	Female sex	1,394 (45.8%)	720 (41.7%)	43.6%	43.1%	0.011
Trial A anti-MRSA	SOFA at infection onset	4.03 (2.13)	4.04 (2.31)	4.04 (2.12)	4.03 (2.33)	0.004
Trial A anti-MRSA	SOFA before time zero	4.92 (3.47)	5.86 (3.98)	5.46 (3.75)	5.51 (3.84)	0.011
Trial A anti-MRSA	Maximum lactate	2.88 (2.51)	3.07 (2.85)	2.99 (2.67)	2.99 (2.76)	0.000
Trial A anti-MRSA	Lactate clearance	20.03 (44.11)	16.08 (56.81)	18.13 (48.60)	18.05 (50.88)	0.002
Trial A anti-MRSA	Minimum MAP	52.35 (12.32)	51.21 (12.34)	51.67 (12.76)	51.70 (12.01)	0.002
Trial A anti-MRSA	Maximum WBC	16.23 (8.45)	17.50 (9.86)	16.90 (9.20)	16.95 (9.06)	0.005
Trial A anti-MRSA	Maximum temperature	37.85 (0.76)	38.03 (0.82)	37.97 (0.81)	37.97 (0.80)	0.002
Trial A anti-MRSA	Initial creatinine	1.47 (1.18)	1.49 (1.19)	1.47 (1.13)	1.49 (1.22)	0.016
Trial A anti-MRSA	Maximum creatinine	1.79 (1.48)	1.78 (1.47)	1.80 (1.43)	1.77 (1.49)	0.021
Trial A anti-MRSA	Urine output	5803.94 (3438.65)	5535.69 (3575.64)	5590.10 (3416.64)	5599.54 (3553.28)	0.003
Trial A anti-MRSA	Vasopressor use	1723 (56.7%)	1,095 (63.4%)	59.0%	61.0%	0.040
Trial A anti-MRSA	Mechanical ventilation	2,264 (74.4%)	1,368 (79.2%)	75.9%	77.7%	0.043
Trial A anti-MRSA	Culture-negative	2,345 (77.1%)	1,355 (78.4%)	77.0%	78.3%	0.029
Trial A anti-MRSA	Recorded non-MDRO positive	696 (22.9%)	373 (21.6%)	23.0%	21.7%	0.029
Trial A anti-MRSA	DNR/DNI	161 (5.3%)	97 (5.6%)	5.5%	5.6%	0.007
Trial B antipseudomonal	Age	65.00 (16.21)	64.26 (15.64)	64.90 (16.22)	64.92 (15.61)	0.002
Trial B antipseudomonal	Female sex	717 (45.7%)	1,354 (44.1%)	45.7%	44.3%	0.029
Trial B antipseudomonal	SOFA at infection onset	3.89 (2.00)	4.12 (2.22)	3.95 (2.03)	3.95 (2.10)	0.002
Trial B antipseudomonal	SOFA before time zero	4.80 (3.33)	5.88 (3.96)	5.07 (3.45)	5.11 (3.58)	0.012
Trial B antipseudomonal	Maximum lactate	2.81 (2.30)	3.11 (2.87)	2.88 (2.38)	2.88 (2.59)	0.001
Trial B antipseudomonal	Lactate clearance	21.27 (36.19)	16.83 (54.15)	20.67 (37.15)	20.20 (41.72)	0.012
Trial B antipseudomonal	Minimum MAP	52.34 (12.58)	51.33 (12.29)	52.03 (12.64)	52.04 (11.97)	0.001
Trial B antipseudomonal	Maximum WBC	15.95 (8.48)	17.67 (10.50)	16.35 (8.77)	16.47 (8.72)	0.013
Trial B antipseudomonal	Maximum temperature	37.84 (0.78)	37.99 (0.82)	37.88 (0.80)	37.89 (0.79)	0.002
Trial B antipseudomonal	Initial creatinine	1.53 (1.18)	1.57 (1.25)	1.53 (1.17)	1.54 (1.25)	0.006
Trial B antipseudomonal	Maximum creatinine	1.81 (1.43)	1.89 (1.53)	1.83 (1.44)	1.82 (1.51)	0.010
Trial B antipseudomonal	Urine output	5737.31 (3640.87)	5276.92 (3466.58)	5575.96 (3559.82)	5582.67 (3511.73)	0.002
Trial B antipseudomonal	Vasopressor use	853 (54.4%)	1896 (61.8%)	56.9%	56.0%	0.018
Trial B antipseudomonal	Mechanical ventilation	1,112 (70.9%)	2,408 (78.4%)	73.3%	73.4%	0.002
Trial B antipseudomonal	Culture-negative	1,181 (75.3%)	2,360 (76.9%)	75.8%	76.0%	0.003
Trial B antipseudomonal	Recorded non-MDRO positive	388 (24.7%)	710 (23.1%)	24.2%	24.0%	0.003
Trial B antipseudomonal	DNR/DNI	93 (5.9%)	185 (6.0%)	6.0%	6.3%	0.012

Values are mean (standard deviation), number (percentage), or percentage unless otherwise indicated. DNR/DNI, do-not-resuscitate/do-not-intubate; MAP, mean arterial pressure; SMD, standardized mean difference; SOFA, Sequential Organ Failure Assessment; WBC, white blood cell count.

### MIMIC-IV primary analysis: antipseudomonal de-escalation

**Figure 3:**
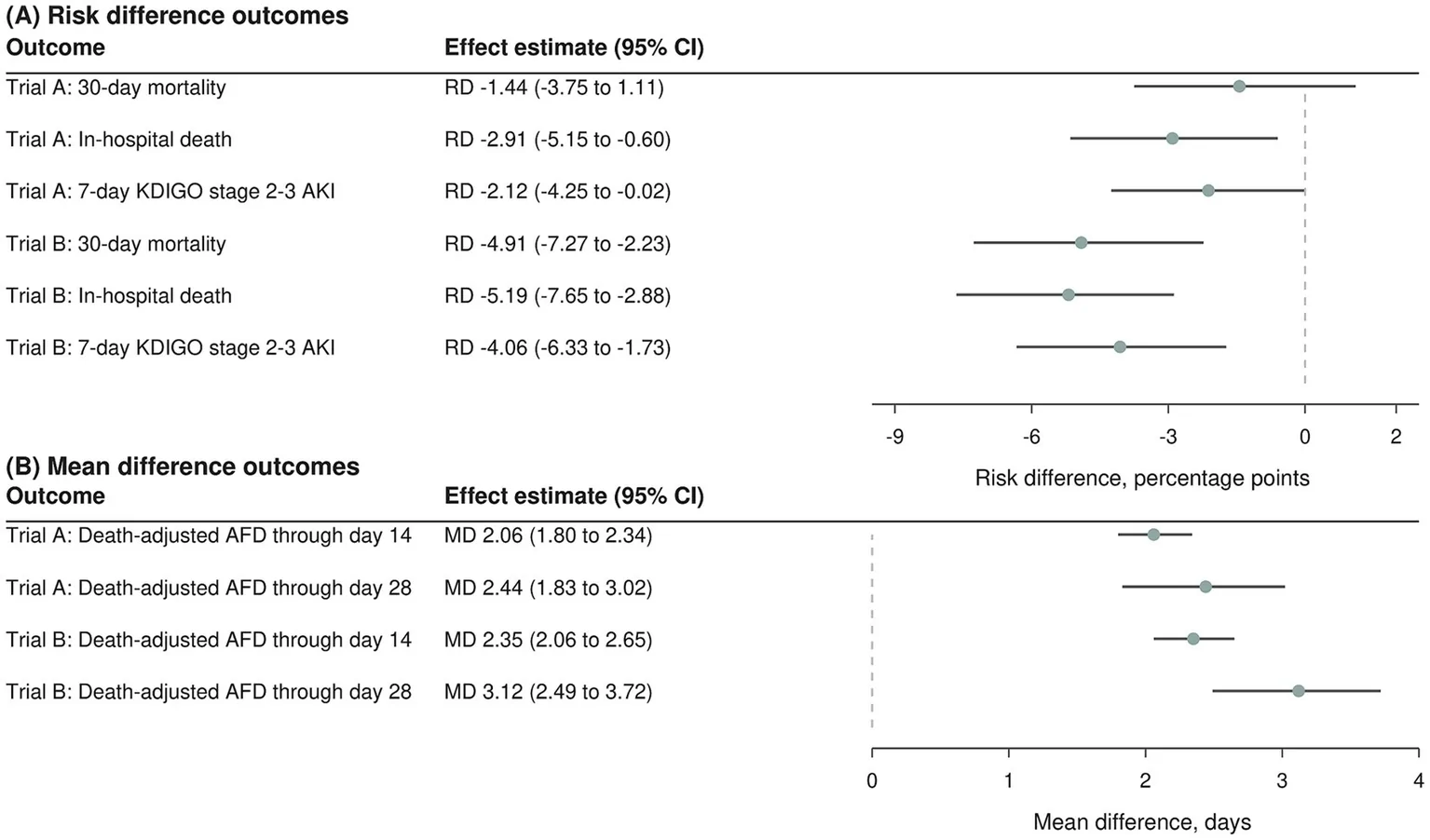
Primary MIMIC-IV outcome estimates after overlap weighting. **(A)** Risk differences for 30-day mortality, in-hospital death, and creatinine-based Kidney Disease: Improving Global Outcomes stage 2–3 acute kidney injury during the 7 days after the landmark. **(B)** Mean differences in death-adjusted antibiotic-free days through days 14 and 28. Estimates of lower mortality and acute kidney injury are exploratory and fall outside the prespecified safety framing. AFD, antibiotic-free day; AKI, acute kidney injury; CI, confidence interval; KDIGO, Kidney Disease: Improving Global Outcomes; MD, mean difference; RD, risk difference.

In Trial B, antipseudomonal de-escalation was associated with lower 30-day mortality than continuation. The overlap-weighted risk was 24.1% in the de-escalated group and 29.0% in the continued group, corresponding to an RD of −4.91 percentage points (95% CI, −7.27 to −2.23) (Table 2; Figure 3). This estimate is presented as an adjusted observational association rather than a causal survival effect. Accordingly, the lower mortality likely reflects prognostic information from the decision to de-escalate (clinicians typically narrow coverage once hemodynamics, organ function, source control, and diagnostic certainty have improved) rather than a survival benefit of the narrower spectrum itself.

**Table 2:** Primary MIMIC-IV outcomes after overlap weighting.

Trial	Outcome	De-escalated	Continued	Effect measure	Effect (95% CI)
Trial A anti-MRSA	30-day mortality	26.3%	27.8%	Risk difference, pp	−1.44 (−3.75 to 1.11)
Trial A anti-MRSA	In-hospital death	20.2%	23.1%	Risk difference, pp	−2.91 (−5.15 to −0.60)
Trial A anti-MRSA	7-day creatinine-based KDIGO stage 2–3 AKI	20.4%	22.5%	Risk difference, pp	−2.12 (−4.25 to −0.02)
Trial A anti-MRSA	Death-adjusted AFD14	7.91 days	5.85 days	Mean difference, days	2.06 (1.80 to 2.34)
Trial A anti-MRSA	Death-adjusted AFD28	17.40 days	14.96 days	Mean difference, days	2.44 (1.83 to 3.02)
Trial B antipseudomonal	30-day mortality	24.1%	29.0%	Risk difference, pp	−4.91 (−7.27 to −2.23)
Trial B antipseudomonal	In-hospital death	17.6%	22.8%	Risk difference, pp	−5.19 (−7.65 to −2.88)
Trial B antipseudomonal	7-day creatinine-based KDIGO stage 2–3 AKI	17.8%	21.8%	Risk difference, pp	−4.06 (−6.33 to −1.73)
Trial B antipseudomonal	Death-adjusted AFD14	8.57 days	6.21 days	Mean difference, days	2.35 (2.06 to 2.65)
Trial B antipseudomonal	Death-adjusted AFD28	18.40 days	15.27 days	Mean difference, days	3.12 (2.49 to 3.72)

Risk differences are reported in percentage points. Mean differences are reported in days. AKI was defined using creatinine-based Kidney Disease: Improving Global Outcomes criteria during the 7 days after the 96 h landmark. AFD14 and AFD28 denote death-adjusted antibiotic-free days through days 14 and 28, respectively. Estimates of lower mortality and acute kidney injury (both trials) fall outside the prespecified safety framing and are exploratory; the prespecified safety interpretation concerns the absence of higher mortality with de-escalation. AFD, antibiotic-free day; AKI, acute kidney injury; CI, confidence interval; KDIGO, Kidney Disease: Improving Global Outcomes.

Antipseudomonal de-escalation was also associated with lower in-hospital death (17.6% vs. 22.8%; RD, −5.19 percentage points; 95% CI, −7.65 to −2.88) and lower 7-day creatinine-based KDIGO stage 2–3 AKI (17.8% vs. 21.8%; RD, −4.06 percentage points; 95% CI, −6.33 to −1.73). Death-adjusted AFDs were higher with de-escalation at day 14 (8.57 vs. 6.21 days; MD, 2.35 days; 95% CI, 2.06 to 2.65) and day 28 (18.40 vs. 15.27 days; MD, 3.12 days; 95% CI, 2.49 to 3.72).

### MIMIC-IV parallel analysis: anti-MRSA de-escalation

In Trial A, anti-MRSA de-escalation showed no evidence of higher 30-day mortality. The overlap-weighted risk was 26.3% in the de-escalated group and 27.8% in the continued group, corresponding to an RD of −1.44 percentage points (95% CI, −3.75 to 1.11).

Anti-MRSA de-escalation was associated with lower in-hospital death (20.2% vs. 23.1%; RD, −2.91 percentage points; 95% CI, −5.15 to −0.60). Seven-day creatinine-based KDIGO stage 2–3 AKI was 20.4% versus 22.5% (RD, −2.12 percentage points; 95% CI, −4.25 to −0.02). Death-adjusted AFDs were higher with de-escalation at day 14 (7.91 vs. 5.85 days; MD, 2.06 days; 95% CI, 1.80 to 2.34) and day 28 (17.40 vs. 14.96 days; MD, 2.44 days; 95% CI, 1.83 to 3.02). Estimates of lower mortality and acute kidney injury fall outside the prespecified safety framing and are exploratory; they should not be interpreted as evidence that de-escalation improved survival or renal outcomes.

### Sensitivity analyses and restart after de-escalation

MIMIC-IV sensitivity analyses were broadly consistent with the primary analysis, particularly for Trial B (Table 3). For antipseudomonal de-escalation, the 30-day mortality RD was −5.63 percentage points (95% CI, −7.92 to −3.18) under a coverage-based exposure definition, −5.18 percentage points (95% CI, −7.74 to −2.66) after excluding final-MDRO-discordant cases, −6.17 percentage points (95% CI, −9.21 to −3.32) in the culture-negative-only cohort, and −4.46 percentage points (95% CI, −6.63 to −1.95) after excluding temperature variables from the propensity score model. In a sensitivity analysis restricted to patients whose relevant microbiology result was recorded before time zero (using the structured result-entry timestamp, storetime, as the strongest available proxy for result availability), all 5,762 strict MIMIC-IV patients were retained; therefore, no patient was reclassified or removed, and the 30-day mortality estimates were unchanged (Trial A RD, −1.44 percentage points [95% CI, −4.13 to 0.67]; Trial B RD, −4.91 percentage points [95% CI, −7.15 to −2.14]); under the storetime proxy, the strict cohort already satisfied result availability at time zero; thus, this confirms the robustness of findings but does not provide an independent removal-based test of look-ahead bias. Among 630 patients excluded for recorded MDRO evidence before time zero, the aggregate MDRO category mapped consistently to the final structured microbiology and susceptibility classification; because this comparison used the same structured source that defined the exclusion, it reflects internal consistency of the structured MDRO flag rather than an organism-level re-adjudication.

**Table 3:** Selected MIMIC-IV sensitivity analyses for 30-day mortality.

Trial	Analysis	*n*	De-escalated	Continued	Effect (95% CI)
Trial A anti-MRSA	Primary analysis	3041/1728	26.3%	27.8%	−1.44 (−3.75 to 1.11)
Trial A anti-MRSA	Coverage-based exposure	3041/2102	26.1%	29.1%	−2.97 (−5.33 to −0.54)
Trial A anti-MRSA	Excluding final MDRO-discordant cases	2875/1554	25.8%	27.6%	−1.82 (−4.13 to 0.76)
Trial A anti-MRSA	Culture-negative only	2345/1355	25.4%	26.6%	−1.18 (−3.92 to 1.58)
Trial A anti-MRSA	Recorded non-MDRO-positive subgroup	696/373	29.2%	31.6%	−2.32 (−7.82 to 3.10)
Trial A anti-MRSA	Propensity score excluding temperature variables	3041/1728	26.5%	27.6%	−1.08 (−3.59 to 1.22)
Trial A anti-MRSA	Microbiology result recorded before time zero	3041/1728	26.3%	27.8%	−1.44 (−4.13 to 0.67)
Trial B antipseudomonal	Primary analysis	1569/3070	24.1%	29.0%	−4.91 (−7.27 to −2.23)
Trial B antipseudomonal	Coverage-based exposure	1569/3273	24.1%	29.7%	−5.63 (−7.92 to −3.18)
Trial B antipseudomonal	Excluding final MDRO-discordant cases	1491/2798	23.8%	29.0%	−5.18 (−7.74 to −2.66)
Trial B antipseudomonal	Culture-negative only	1181/2360	22.9%	29.1%	−6.17 (−9.21 to −3.32)
Trial B antipseudomonal	Recorded non-MDRO-positive subgroup	388/710	27.8%	28.7%	−0.93 (−6.62 to 4.28)
Trial B antipseudomonal	Propensity score excluding temperature variables	1569/3070	24.2%	28.7%	−4.46 (−6.63 to −1.95)
Trial B antipseudomonal	Microbiology result recorded before time zero	1569/3070	24.1%	29.0%	−4.91 (−7.15 to −2.14)

The n column shows de-escalated/continued patients. Effect estimates are risk differences in percentage points. In the “Microbiology result recorded before time zero” sensitivity analysis, the maximum weighted absolute SMD was 0.101 for Trial A; all other covariates and analyses had maximum SMD below 0.10. CI, confidence interval; MDRO, multidrug-resistant organism.

Same-class restart after de-escalation was described only among de-escalated patients. Anti-MRSA restart between 48 h and 7 days after the analysis start occurred in 287 of 3,041 de-escalated Trial A patients (9.4%; Wilson 95% CI, 8.4 to 10.5%) at a median of 3.50 days after the 96-h landmark (IQR, 2.67 to 4.88); antipseudomonal restart occurred in 157 of 1,569 de-escalated Trial B patients (10.0%; Wilson 95% CI, 8.6 to 11.6%) at a median of 3.33 days (IQR, 2.50 to 4.50). Restarted patients had higher 30-day mortality than non-restarted de-escalated patients (Trial A, 34.8% vs. 23.1%; Trial B, 29.9% vs. 22.8%) and higher in-hospital mortality (Trial A, 32.8% vs. 16.5%; Trial B, 26.8% vs. 15.7%), consistent with restart being a marker of clinical deterioration in a subset. Because structured data did not reliably capture the reason for restart, it is reported as a descriptive safety indicator and was not compared with continuation or treated as a treatment-failure endpoint (Supplementary Table 2).

### eICU-CRD supportive multicenter analysis

**Figure 4:**
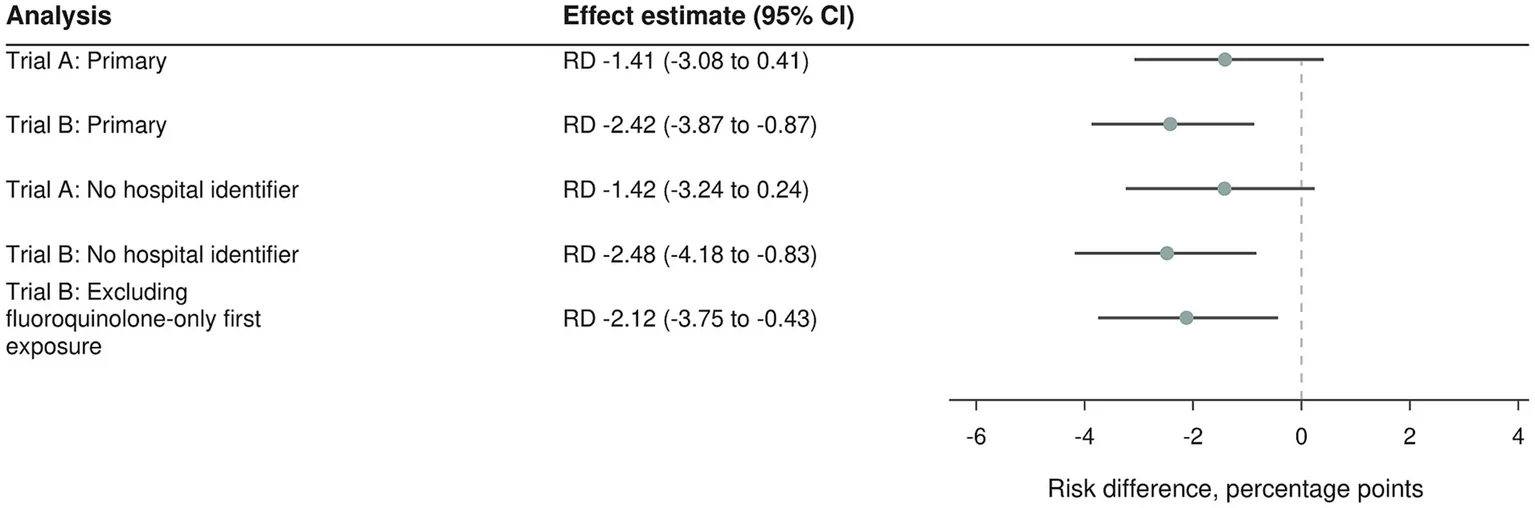
eICU-CRD supportive multicenter analysis of hospital mortality. Primary estimates use hospital-cluster bootstrap confidence intervals; sensitivity analyses omit hospital identifiers or exclude fluoroquinolone-only first exposure. CI, confidence interval; MRSA, methicillin-resistant *Staphylococcus aureus*; RD, risk difference.

In the eICU-CRD supportive multicenter analysis, Trial B showed a hospital-mortality association in the same direction as the MIMIC-IV estimate. The hospital-cluster bootstrap was the primary uncertainty estimate. Weighted hospital mortality was 10.2% in the de-escalated group and 12.7% in the continued group, corresponding to an RD of −2.42 percentage points (95% CI, −3.87 to −0.87) and a risk ratio of 0.81 (95% CI, 0.70 to 0.93) (Table 4; Figure 4). The full set of eICU-CRD estimates is shown in Supplementary Figure 1. Covariate balance and propensity score distributions are shown in Supplementary Figures 2–5. Leave-one-hospital-out estimates for Trial B are shown in Supplementary Figure 6 and ranged from approximately −2.66 to −2.19 percentage points.

**Table 4:** eICU-CRD supportive multicenter analysis and hospital-level sensitivity analyses.

Trial	Analysis	*N*	*n* de-escalated/continued	De-escalated mortality	Continued mortality	RD, pp (95% CI)	RR (95% CI)
Trial A anti-MRSA	Primary	5,360	3866/1494	10.7%	12.1%	−1.41 (−3.08 to 0.41)	0.88 (0.76 to 1.04)
Trial B antipseudomonal	Primary	5,439	2678/2761	10.2%	12.7%	−2.42 (−3.87 to −0.87)	0.81 (0.70 to 0.93)
Trial A anti-MRSA	No hospital identifier	5,360	3866/1494	10.7%	12.1%	−1.42 (−3.24 to 0.24)	0.88 (0.76 to 1.02)
Trial B antipseudomonal	No hospital identifier	5,439	2678/2761	10.2%	12.7%	−2.48 (−4.18 to −0.83)	0.81 (0.69 to 0.93)
Trial B antipseudomonal	Excluding fluoroquinolone-only first exposure	4,815	2373/2442	10.5%	12.6%	−2.12 (−3.75 to −0.43)	0.83 (0.73 to 0.96)

For primary rows, uncertainty was estimated with hospital-cluster bootstrap. eICU-CRD was used to assess directional consistency in a broader multicenter cohort rather than to formally validate the strict MIMIC-IV no-MDRO cohort; missing microLab information was not interpreted as culture-negative. CI, confidence interval; RD, risk difference; RR, risk ratio.

For Trial A in eICU-CRD, weighted hospital mortality was 10.7% with de-escalation and 12.1% with continuation. The cluster-bootstrap RD was −1.41 percentage points (95% CI, −3.08 to 0.41), with a risk ratio of 0.88 (95% CI, 0.76 to 1.04).

## Discussion

In this target trial emulation of ICU patients with sepsis who reached a day-4 reassessment window, de-escalation was not associated with higher mortality and was consistently associated with more death-adjusted AFDs. Trial B also showed lower 30-day mortality, in-hospital death, and 7-day creatinine-based KDIGO stage 2–3 AKI in MIMIC-IV, while the eICU-CRD supportive analysis produced a hospital-mortality estimate in the same direction. In Trial A, there was no evidence of higher 30-day mortality, and antibiotic exposure was lower. The most defensible interpretation is therefore one of safety and reduced exposure. The lower mortality and AKI estimates, particularly in Trial B, are exploratory and should not be interpreted as showing that de-escalation itself improved survival or renal outcomes.

The contrast with the community-onset sepsis target trial emulation by Gupta et al. requires explicit consideration. In 36,924 patients, day-4 anti-MRSA and antipseudomonal de-escalation were associated with similar 90-day mortality to continuation (anti-MRSA odds ratio, 1.00; 95% CI, 0.88–1.14; antipseudomonal odds ratio, 0.98; 95% CI, 0.86–1.13), together with fewer antibiotic days and shorter hospitalization (5). The larger mortality association in our ICU cohort is unlikely to establish a biological survival benefit from narrowing antibiotics. This pattern is best understood as confounding by indication. A more plausible explanation is greater residual confounding by early clinical recovery in the ICU: clinicians may de-escalate when hemodynamics, organ function, source control, and diagnostic certainty have improved, and this bedside gestalt is only partly represented by structured variables. The ICU-specific contribution of the present study is therefore not evidence of mortality benefit, but an evaluation of a common landmark decision with detailed severity trajectories, AKI and AFD outcomes, and a supportive multicenter analysis.

The timing of the analysis remains an important design strength. Studies that classify broad-spectrum exposure over the course of an admission can be vulnerable to time-related bias and may not represent an actual stewardship decision. Here, time zero was set at 72 h after broad-spectrum antibiotic initiation, exposure was classified during a 24 h grace period, and follow-up began at 96 h. This design separated treatment classification from outcome follow-up and defined the target population as patients who were alive and remained under care at the day-4 decision point. The day-4 timing corresponds to a common ICU reassessment point at which early culture information, absence of recorded MDRO evidence, hemodynamic trajectory, laboratory trends, and source-control status are reviewed together. Accordingly, these findings apply specifically to structured day-4 reassessment decisions and should not be interpreted as identifying the earliest possible opportunity for antibiotic narrowing. In routine practice, de-escalation may be prompted by negative or non-MDRO cultures, narrowing of the suspected infection syndrome, clinical stabilization, improving organ function, or a combination of these factors. These reasons could not be reliably separated in structured EHR data, and their incomplete measurement is a likely source of residual confounding by indication.

The stronger Trial B association may also reflect the clinical heterogeneity of antipseudomonal treatment. De-escalation can reduce exposure to broad beta-lactams and combination regimens, but it can also be a marker of recovery, adequate source control, or greater diagnostic clarity. ICU studies and expert statements support narrowing therapy when the clinical and microbiological evidence permits, while cautioning against indiscriminate discontinuation in unstable patients (19–21). The present results are consistent with that stewardship principle, but they do not distinguish the effect of a reduced spectrum from the prognostic information contained in the decision to de-escalate.

The kidney findings require similar restraint. Randomized and observational evidence has focused attention on renal outcomes during broad beta-lactam and vancomycin-containing therapy (22, 23). Our endpoint was creatinine-based KDIGO stage 2–3 AKI during the 7 days after the landmark, and death was not modeled as a competing event. Because mortality was higher in the continuation group, failure to model death as a competing event would be expected to reduce observed AKI incidence in that group by removing some high-risk patients from follow-up; this mechanism would tend to attenuate, rather than exaggerate, the lower AKI estimate observed with de-escalation. Because mortality was lower in the de-escalated group, earlier death alone is unlikely to explain its lower observed AKI risk; however, discharge, measurement intensity, pre-existing renal trajectory, and unmeasured recovery may continue to influence the estimate. The AKI result should therefore be regarded as hypothesis-generating rather than evidence of a specific drug-toxicity pathway.

The findings also fit a wider movement toward shorter and more individualized antibiotic courses in severe infection. Biomarker-guided strategies, randomized trials of bloodstream infection duration, and recent meta-analyses have shown that less antibiotic exposure can be appropriate in selected patients when clinical stability and source control are present (24–29). Our study addresses a related but distinct question: whether unnecessary resistant-pathogen coverage can be narrowed at day 4 while appropriate narrower treatment continues.

Implementation should be cautious and auditable. Stewardship guidance supports a systematic review of indication, spectrum, route, and duration, while guidance for antimicrobial-resistant gram-negative infections emphasizes matching spectrum to microbiological evidence rather than continuing the broadest empiric agent by default (30, 31). Critical-care reviews similarly describe de-escalation as a clinical process that combines microbiology, physiologic trajectory, and source-control assessment (32, 33).

At the bedside, the results support a structured day-4 review rather than automatic discontinuation. Clinicians should ask whether MDRO evidence has emerged, whether the patient has stabilized, whether source control is adequate, and whether continued antipseudomonal or anti-MRSA coverage remains justified. The findings should not be extrapolated to patients with persistent shock, uncontrolled infection, severe immunosuppression, chronic dialysis, or infections that generally require prolonged treatment. They also do not imply that culture-negative sepsis is non-infectious. The narrower conclusion is that, among selected patients without a clear indication for continued resistant-pathogen coverage, de-escalation was not associated with worse measured outcomes and reduced antibiotic exposure.

The eICU-CRD analysis should be read as supportive rather than confirmatory. Its microLab table is sparse and heterogeneous across hospitals; therefore, the strict MIMIC-IV culture-availability and no-MDRO criteria could not be reproduced. Missing microbiology data were not interpreted as negative cultures. Absolute mortality risks were lower in eICU-CRD and are not directly comparable with MIMIC-IV because the eICU-CRD analysis used hospital mortality in a broader union cohort, whereas MIMIC-IV used 30-day mortality in a strict no-MDRO landmark cohort. The value of eICU-CRD is limited to directional consistency and hospital-level robustness but not formal validation or evidence of a transportable causal effect.

This study has limitations. First, treatment was not randomized. Despite detailed early severity variables, overlap weighting, and good measured balance, residual confounding by clinical improvement, source-control confidence, and treatment selection is likely and may account for part of the Trial B mortality and AKI associations. Second, microbiology reporting time could not always be observed directly. We used the structured result-entry timestamp (storetime) as the strongest available proxy for result availability; a restricted sensitivity analysis retained all patients and left the mortality estimates unchanged. However, storetime may not perfectly reproduce what clinicians knew at the bedside at 72 h; therefore, residual look-ahead bias cannot be fully excluded if final results were recorded after the day-4 decision. Third, infection-source classification was not available through a validated framework in the frozen dataset. Although diagnosis codes, specimen descriptions, and microbiology records were available, reconstructing source categories from these elements could introduce additional misclassification. Therefore, we could not fully assess whether de-escalation decisions differed across infection syndromes. Fourth, the strict MIMIC-IV cohort excluded patients without pre-time-zero culture information, patients receiving chronic dialysis, those with severe immunosuppression, and those with fluoroquinolone-only initial broad-spectrum exposure, which narrows generalizability. Fifth, death was not handled with a formal competing-risk model for AKI, and discharge or differential laboratory measurement could affect renal-event ascertainment. Sixth, the eICU-CRD analysis was supportive rather than a strict external validation because microbiology and medication completeness varied across hospitals. Seventh, bootstrap uncertainty estimates were based on 500 MIMIC-IV and 300 eICU-CRD replicates and may therefore contain some Monte Carlo variation. Finally, antibiotic restart was descriptive within de-escalated patients and was not a comparative treatment-failure endpoint.

A pragmatic ICU stewardship trial is the appropriate next step. Such a trial could use the same day-4 eligibility logic; require the explicit assessment of MDRO evidence, clinical stability, and source control; and randomize or cluster-randomize a de-escalation recommendation against usual care. Mortality safety, antibiotic exposure, AKI, and treatment re-escalation should be prespecified, with adequate power for noninferiority rather than an expectation of survival benefit.

## Conclusion

Among ICU patients with sepsis who survived to a day-4 reassessment window, antipseudomonal and anti-MRSA de-escalation showed no evidence of higher mortality and were associated with more death-adjusted antibiotic-free days (AFDs). Lower mortality and AKI estimates in Trial B were exploratory and may reflect residual confounding by early recovery and treatment selection. The eICU-CRD analysis provided directional multicenter support but was not a formal external validation of the strict MIMIC-IV cohort. These findings justify prospective evaluation of structured day-4 de-escalation strategies, with mortality framed as a safety outcome rather than an anticipated benefit.

## Data Availability

The datasets analyzed in this study are publicly available to credentialed researchers from PhysioNet. MIMIC-IV (v3.1) can be accessed at https://physionet.org/content/mimiciv/ and the eICU Collaborative Research Database (v2.0) at https://physionet.org/content/eicu-crd/, subject to completion of the required training and a data use agreement.
