# Supplementary material for: Day-4 antipseudomonal and anti-MRSA de-escalation was not associated with higher mortality in critically ill patients with sepsis
Source: Front Med (Lausanne). 2026 Aug 27;13:1924203. doi: 10.3389/fmed.2026.1924203 (PMC13558194; doi:10.3389/fmed.2026.1924203)
Supplement: Supplementary file 1 [file Supplementary_file_1.DOCX]

**Supplementary Material**

**Day-4 antipseudomonal and anti-MRSA de-escalation was not associated with higher mortality in critically ill patients with sepsis**

Yuhang Zhang, Guoying Liao, Jianli Song, Yinsheng Liao, Lulu Zhang

**Contents**

| Supplementary Figure 1. eICU-CRD supportive multicenter analysis and sensitivity analyses for hospital mortality | 2 |
| --- | --- |
| Supplementary Figure 2. eICU-CRD Trial A covariate balance without hospital identifiers | 3 |
| Supplementary Figure 3. eICU-CRD Trial B covariate balance without hospital identifiers | 4 |
| Supplementary Figure 4. Propensity score distribution in Trial A | 5 |
| Supplementary Figure 5. Propensity score distribution in Trial B | 6 |
| Supplementary Figure 6. Leave-one-hospital-out sensitivity analysis for Trial B | 7 |
| Supplementary Table 1. Early antibiotic-class pattern, culture status, and comorbidities in the MIMIC-IV strict cohort | 8 |
| Supplementary Table 2. Same-class restart timing and mortality among de-escalated patients  Supplementary Table 3. Time-zero-visible microbiological characteristics and effective sample size after overlap weighting in the MIMIC-IV strict landmark cohort 10 | 9 |
|  |  |

Supplementary Table 3. Time-zero-visible microbiological characteristics and effective sample size after overlap weighting in the MIMIC-IV strict landmark cohort

**Supplementary Figure 1. eICU-CRD supportive multicenter analysis and sensitivity analyses for hospital mortality.** The forest plot shows risk differences in hospital mortality, in percentage points, for Trial A (anti-MRSA de-escalation) and Trial B (antipseudomonal de-escalation). Primary estimates used hospital-cluster bootstrap confidence intervals. Sensitivity analyses omitted hospital identifiers, restricted to patients still in an intensive care unit at 96 h or 72 h where shown, or excluded fluoroquinolone-only first exposure. The dashed vertical line at 0 indicates no difference between de-escalation and continuation. CI, confidence interval; eICU-CRD, eICU Collaborative Research Database; ICU, intensive care unit; MRSA, methicillin-resistant Staphylococcus aureus; RD, risk difference.

**Supplementary Figure 2. Covariate balance in the eICU-CRD Trial A model without hospital identifiers.** The Love plot shows absolute standardized mean differences before and after overlap weighting for the anti-MRSA de-escalation target trial. Each row represents a baseline or early-treatment covariate. Purple dots denote covariate imbalance before weighting, and green dots denote imbalance after overlap weighting. The dashed vertical line marks the prespecified balance threshold of 0.10. APACHE, Acute Physiology and Chronic Health Evaluation; CSICU, cardiac surgery intensive care unit; ICU, intensive care unit; MICU, medical intensive care unit; MRSA, methicillin-resistant Staphylococcus aureus; PACU, post-anesthesia care unit; SDU, step-down unit; SICU, surgical intensive care unit; SMD, standardized mean difference.

**Supplementary Figure 3. Covariate balance in the eICU-CRD Trial B model without hospital identifiers.** The Love plot shows absolute standardized mean differences before and after overlap weighting for the antipseudomonal de-escalation target trial. Each row represents a baseline or early-treatment covariate. Purple dots denote covariate imbalance before weighting, and green dots denote imbalance after overlap weighting. The dashed vertical line marks the prespecified balance threshold of 0.10. APACHE, Acute Physiology and Chronic Health Evaluation; CSICU, cardiac surgery intensive care unit; ICU, intensive care unit; MICU, medical intensive care unit; PACU, post-anesthesia care unit; SICU, surgical intensive care unit; SMD, standardized mean difference; WBC, white blood cell count.

**Supplementary Figure 4. Propensity score distribution for de-escalation in Trial A.** The density plot shows the estimated propensity score for receiving anti-MRSA de-escalation in the eICU-CRD supportive multicenter analysis. The green line represents de-escalated patients, and the orange line represents patients who continued anti-MRSA coverage. Overlap between the curves indicates the region of clinical equipoise emphasized by overlap weighting. eICU-CRD, eICU Collaborative Research Database; MRSA, methicillin-resistant Staphylococcus aureus.

**Supplementary Figure 5. Propensity score distribution for de-escalation in Trial B.** The density plot shows the estimated propensity score for receiving antipseudomonal de-escalation in the eICU-CRD supportive multicenter analysis. The green line represents de-escalated patients, and the orange line represents patients who continued antipseudomonal coverage. Overlap between the curves indicates the region of clinical equipoise emphasized by overlap weighting. eICU-CRD, eICU Collaborative Research Database.

**Supplementary Figure 6. Leave-one-hospital-out sensitivity analysis for Trial B.** Each point shows the risk difference in hospital mortality, in percentage points, for the antipseudomonal de-escalation target trial after excluding the hospital listed on the y-axis from the eICU-CRD supportive multicenter analysis. The dashed vertical line at 0 indicates no difference between de-escalation and continuation. The estimates remained directionally consistent across leave-one-hospital-out analyses, indicating that the Trial B supportive analysis was not driven by a single hospital. eICU-CRD, eICU Collaborative Research Database; RD, risk difference.

**Supplementary Table 1. Early antibiotic-class pattern, pre-time-zero culture status, and major comorbidities in the MIMIC-IV strict cohort (n = 5,762).**

| **Characteristic** | **No. (%)** |
| --- | --- |
| ***Early broad-spectrum antibiotic-class pattern*** | |
| Anti-MRSA only | 1301 (22.6) |
| Antipseudomonal only | 1502 (26.1) |
| Combined anti-MRSA and antipseudomonal | 2959 (51.4) |
| ***Pre-time-zero culture status*** | |
| Culture-negative | 4450 (77.2) |
| Recorded non-MDRO positive | 1312 (22.8) |
| ***Major comorbidities (Quan Charlson, index admission)*** | |
| Diabetes mellitus | 1860 (32.3) |
| Congestive heart failure | 2183 (37.9) |
| Chronic pulmonary disease | 1868 (32.4) |
| Chronic kidney disease | 1235 (21.4) |
| Chronic liver disease | 1126 (19.5) |
| Prior myocardial infarction | 1122 (19.5) |
| Cerebrovascular disease | 778 (13.5) |
| Malignancy (including metastatic) | 772 (13.4) |
| Peripheral vascular disease | 703 (12.2) |
| Dementia | 268 (4.7) |

Values are No. (%). Comorbidities were derived from index-hospitalization diagnosis codes mapped to Quan Charlson domains (Quan H et al. Med Care. 2005;43:1130–1139); diabetes, liver disease, and malignancy combine their respective severity subdomains. The prior-myocardial-infarction row reflects the Charlson myocardial-infarction domain only and does not include stable coronary artery disease. Two hospitalizations had no recorded diagnosis codes and contribute to the denominator only. The early antibiotic-class pattern reflects coverage received during early treatment and is a different partition from the trial eligibility shown in Figure 1. In eICU-CRD, comorbidity and antibiotic-class fields were not available in comparable form, and microbiology (microLab) records were present for only 97 of 9,487 encounters (1.0%); missing records were not interpreted as culture-negative, consistent with the supportive-only role of that cohort. MDRO, multidrug-resistant organism; MRSA, methicillin-resistant Staphylococcus aureus.

**Supplementary Table 2. Same-class restart timing and mortality among de-escalated patients.**

Supplementary Table 3. Time-zero-visible microbiological characteristics and effective sample size after overlap weighting in the MIMIC-IV strict landmark cohort.

| **Measure** | **Trial A (anti-MRSA de-escalation)** | **Trial B (antipseudomonal de-escalation)** |
| --- | --- | --- |
| De-escalated patients, No. | 3,041 | 1,569 |
| Same-class restart, No. (%) | 287 (9.4) | 157 (10.0) |
| Median time to restart, days (IQR) | 3.50 (2.67–4.88) | 3.33 (2.50–4.50) |
| 30-day mortality, restarted, No./total (%) | 100/287 (34.8) | 47/157 (29.9) |
| 30-day mortality, not restarted, No./total (%) | 637/2,754 (23.1) | 322/1,412 (22.8) |
| In-hospital mortality, restarted, No./total (%) | 94/287 (32.8) | 42/157 (26.8) |
| In-hospital mortality, not restarted, No./total (%) | 454/2,754 (16.5) | 221/1,412 (15.7) |

Restart was defined as resumption of same-class coverage between 48 hours and 7 days after the analysis start, among de-escalated patients only. Mortality comparisons are within the de-escalated group (restarted vs not restarted) and were not made against the continuation group; restart is reported as a descriptive safety indicator rather than a treatment-failure endpoint. Structured data did not reliably capture the clinical reason for restart. IQR, interquartile range; MRSA, methicillin-resistant Staphylococcus aureus.

# Supplementary Table 3. Time-zero-visible microbiological characteristics and effective sample size after overlap weighting in the MIMIC-IV strict landmark cohort.

## A. Infection-source classification availability

| Variable | Status |
| --- | --- |
| Validated infection-source classification | Not available in the frozen analytical dataset. |
| Rationale | Diagnosis codes, specimen descriptions, and microbiology records were available; however, post hoc infection-source reconstruction was not performed because it could introduce misclassification. |

## B. Time-zero-visible microbiological characteristics

| Characteristic | No. (%) / Source |
| --- | --- |
| Culture-negative | 4450 (77.2) |
| Any positive culture available by time zero | 1312 (22.8) |
| Gram-positive organisms | See Supplementary Data File: organism_distribution_mimic_strict.csv |
| Gram-negative organisms | See Supplementary Data File: organism_distribution_mimic_strict.csv |
| Polymicrobial cultures | See Supplementary Data File: organism_distribution_mimic_strict.csv |
| Methicillin-resistant Staphylococcus aureus (MRSA) | See Supplementary Data File: organism_distribution_mimic_strict.csv |
| Pseudomonas aeruginosa | See Supplementary Data File: organism_distribution_mimic_strict.csv |
| Enterobacterales | See Supplementary Data File: organism_distribution_mimic_strict.csv |

## C. Effective sample size after overlap weighting

| Trial | Strategy | Unweighted n | Weighted ESS |
| --- | --- | --- | --- |
| Trial A anti-MRSA | De-escalated | 3041 | 2798.9 |
| Trial A anti-MRSA | Continued | 1728 | 1679.8 |
| Trial B antipseudomonal | De-escalated | 1569 | 1542.0 |
| Trial B antipseudomonal | Continued | 3070 | 2841.9 |

ESS was calculated using the final overlap weights: ESS = (Σw)² / Σw². This supplementary analysis was descriptive and did not alter cohort definitions, exposure classification, weighting strategy, or primary outcome analyses.
